# Prevalence of abortion and adverse pregnancy outcomes among working women in Korea: A cross-sectional study

**DOI:** 10.1371/journal.pone.0182341

**Published:** 2017-08-29

**Authors:** Chulyong Park, Mo-Yeol Kang, Dohyung Kim, Jaechan Park, Huisu Eom, Eun-A Kim

**Affiliations:** 1 Occupational Safety and Health Research Institute, Korea Occupational Safety and Health Agency, Ulsan, Korea; 2 Department of Occupational and Environmental Medicine, Sungkyunkwan University School of Medicine, Seoul, Korea; 3 Department of Preventive Medicine, Seoul National University College of Medicine, Seoul, Korea; University of the West Indies Faculty of Medical Sciences Mona, JAMAICA

## Abstract

**Objectives:**

To investigate incidence and distribution of major adverse reproductive health problems related to various kinds of industries in Korea and to compare risks for major reproductive outcomes to assess maternal health in working and non-working women.

**Methods:**

We requested claim data from the Korean National Health Insurance. We defined reference groups as (1) non-working women and (2) workers in the education field. Women working in each industry were compared with reference groups regarding rates of miscarriage, threatened abortion, preterm labor, and intrauterine growth restriction. Logistic regression was used for multivariate analysis, and age and income adjustment was performed.

**Results:**

The percentages of all adverse obstetric outcomes were higher in working women than in non-working women. Working women had higher and statistically significant adjusted odds ratios (ORs) for miscarriage in 18 of the 21 industries. The age and income-adjusted OR for miscarriage for all working women was 1.26 (95% confidence interval, 1.23–1.28). Business facilities management and business support services, manufacturing, human health and social work activities, wholesale and retail trade, and professional, scientific, and technical activities were major industries with higher adjusted ORs for adverse obstetric outcomes.

**Conclusions:**

We confirmed that compared to non-working women, working women have a higher risk for adverse pregnancy outcomes. Thus, adverse pregnancy outcomes such as threatened abortion, preterm labor, and intrauterine growth restriction may be associated with working status. This exploratory study identified several industries where in-depth studies are required in future to improve occupational safety in women of reproductive age.

## Introduction

Reproductive health is an important issue, especially in women of childbearing age. Women in modern Korean society start working during their mid-20s or early 30s, and this time period overlaps with marriage, pregnancy, and delivery. Over time, the employment rate for women has increased and work environments have changed. The increased number of working women increases the chance that women could be exposed to various occupational hazards during pregnancy. Various occupational reprotoxic agents, including chemicals, physical agents, ergonomic factors, and suspicious agents, have been examined and suggested to negatively affect the reproductive health of women [1–3]. In addition to these factors, work hours, shift work, and job stress have been suggested to affect the reproductive outcomes of women [4–6], and there are still unknown reprotoxic agents.

Abortion is one relatively common type of pregnancy termination. Abortion rates are different depending on country, age, and race; however, 3 of 10 women experience abortion during their lifetime [7]. The International Classification of Diseases, Tenth Revision (ICD-10) categorizes abortion as ectopic pregnancy, hydatidiform mole, other abnormal products of conception, spontaneous abortion (miscarriage), medical abortion, other abortion, and unspecified abortion. Among these, missed abortion (missed miscarriage) and spontaneous abortion (miscarriage) comprise most abortions that can be attributable to various causes, including occupational factors. Other adverse reproductive complications such as preterm labor, fetal abnormality, and intrauterine growth restriction (IUGR) have also been studied to shed light on their relation to occupation and environmental factors.

There is an ongoing demand to elucidate the scale, effects, and mechanisms of action of reprotoxic factors and to elucidate epidemiological traits of adverse reproductive health. However, it is difficult to identify and quantify a single agent’s effect because human development and reproduction are complicated. They interact with various conditions, including individual traits. In addition to individual traits, occupational causes are important factors to be considered, because many reprotoxic agents can be found at work; those who do work spend most of their time at their workplaces. Several occupations and their relationship with the reproductive health of working women have been studied, such as nurses [8–11], hair stylists [12–19], flight attendants [20–23], plastic manufacturers [24–27], and semiconductor workers [28–33]. Studies confined to a specific occupation or industry could elucidate detailed causal relationships; however, the overall reproductive risk of working women in various workplaces could not be shown. In addition, the risk ratios of workplace reproductive hazards in a specific study cannot be compared directly because of differences in the study population and design. There are a few reproductive hazard-related studies that were performed using nationwide data to compare several types of industries in an attempt to provide scientific proof for the necessity of public preventative policies.

Our study aimed to provide incidence and distribution of major adverse reproductive health problems such as miscarriage, threatened abortion, preterm labor, and IUGR related to industrial work in Korea. We also focused on the neglected industries in terms of maternal health and the provisions for preventative management.

## Materials and methods

### Study populations

We included a study population of Korean National Health Insurance (NHI) holders in 2013. We requested the NHI claim data of all insured women diagnosed as being pregnant, having a child, and being in puerperium (ICD-10 codes O00-O99). NHI is a medical insurance program overseen by the Korean government that covers more than 97% of the Korean population. There are three types of insurance holders: insured employees (workplace insurance), insured self-employed (community insurance), and medical aid beneficiaries [34]. Those who are insured can have their dependents, such as the insured person’s spouse, direct lineal descendants, and unmarried brothers or sisters who do not have incomes, insured. The dependents receive insurance benefits without paying their own insurance fee; their fee is included in the insurance fee of their spouse or parent. We assumed that the dependents did not work and were not economically active.

We defined the reference groups as non-working women and workers in the education field. Non-working women are free from occupational factors that may negatively affect their reproductive health. Likewise, education workers are generally not involved in shift work or exposed to reprotoxic agents. Therefore, we considered them as the second reference group. From the 2013 NHI claim data, we found 430,343 pregnancies and 340,088 deliveries. In Korea, 436,455 babies were born in 2013 and thus, our data comprise 77.9% of these births. We excluded the community insurance group, which comprises of individuals who have their own businesses or do not work at usual workplaces included in this study. We also excluded medical beneficiaries because their socio-economic status and work characteristics are different from those of our study groups.

### Study variables

To define obstetric outcomes, we requested diagnostic codes of the NHI claim data. The NHI follows the diagnostic codes and descriptions of ICD-10. There are codes for prenatal care (Z32, Z33, and Z34), but not all hospitals use these codes. For a more inclusive study design, we tried to obtain all possible pregnancy cases. Since all pregnancies end in either delivery or abortion, we defined pregnancy as all insured women who experienced delivery or abortion. In addition, we excluded all other forms of abortions, such as medical abortion (O04), other abortion (O05), and unspecified abortion (O06), to rule out abortion outcomes that could not be attributable to occupational and environmental factors. We also excluded stillbirth (P95), which is a rare outcome of pregnancy that does not belong with the puerperium codes. We only included missed abortion (O021) and spontaneous abortion (O03), both of which were defined as miscarriage. All definitions and codes of obstetric outcomes and complications used in this study are shown in Table 1.

**Table 1:** Definition of abortion, delivery, pregnancy, and obstetric outcomes.

Variables	ICD-10 code
Abortion Missed abortion Spontaneous abortion	O00-O06 (O00-O069) O021 O03
Delivery	O80-O84
Pregnancy	Any insured woman who experienced abortion or delivery
Miscarriage	Any insured woman diagnosed as having a missed abortion or spontaneous abortion
Threatened abortion	O200
Preterm labor	O60 (O600, O601, O602, O603)
IUGR	O365

ICD-10, International Classification of Diseases, Tenth Revision; IUGR, intrauterine growth restriction

We obtained basic information of the study population from the qualification data of NHI claims, which contain information regarding sex, age, insurance fee premium, and occupational industry. NHI insurance fee premiums have 20 classes that are based on incomes of the insurance holders. Each class has the same population; insurance fee premiums class 1 is for those earning the lowest 5% of all incomes, and insurance fee premiums class 20 is for those earning the top 5% of all incomes. We consider insurance fee premiums to be proxy indicators of the socio-economic status of the study groups. Industrial classification codes are also provided in the qualification data; these follow the Korean Standard Industrial Classification codes. Korean Standard Industrial Classification categories for industry, group, and class are based on the International Standard Industrial Classification of the United Nations.

### Statistical analysis

We classified our study populations into 21 industries and calculated the numbers of pregnancies, abortions, threatened abortions, and other obstetric complications. A frequency analysis was performed for general characteristics and numbers of each case. The mean values (±standard deviation) of age and income for all patients with pregnancy in each group are presented. Regarding data analysis, we assumed that all study populations could be diagnosed as having a specific obstetric code only once. For example, we considered one’s repetitive missed abortions as one missed abortion. Additionally, we excluded multiple insurance claims with the same diagnostic codes. Logistic regression was used for multivariate analysis, and age and income adjustments were performed. SAS version 9.3 (SAS Institute) was used for all analyses.

### Ethics statements

Our study was approved by the Institutional Review Board at the Occupational Safety and Health Research Institute of Korea Occupational Safety and Health Agency (OSHRI-2015-06). The NHI provided all data with randomized and encoded identification numbers and thus, it was impossible to link the data with respective individuals or to other data.

## Results

The numbers of pregnancies, obstetric outcomes, and general characteristics of each field are shown in Table 2.

**Table 2:** General characteristics of the study population and obstetric outcomes.

Industry	Pregnancy	Age (Mean±SD)	Income (Mean±SD)	Obstetric outcomes						
				Delivery (%)	Abortion (%)	Missed abortion (%)	Spontaneous abortion (%)	Threatened abortion (%)	Preterm labor (%)	IUGR (%)
**Dependents of those with employment-based NHI** **(non-working women)**	219,767	31.12 (±4.46)	12.12 (±4.88)	177,887 (80.9)	41,942 (19.1)	27,218 (12.4)	6,925 (3.2)	27,426 (12.5)	23,289 (10.6)	3,253 (1.5)
**All working women**	210,576	31.09 (±3.94)	9.90 (±4.77)	162,201 (77.0)	48,454 (23.0)	31,391 (14.9)	8,572 (4.1)	36,229 (17.2)	23,905 (11.4)	3,473 (1.6)
Agriculture, forestry, and fishing	402	31.13 (±4.21)	7.97 (±4.68)	291 (72.4)	111 (27.6)	73 (18.2)	15 (3.7)	60 (14.9)	37 (9.2)	3 (0.7)
Mining and quarrying	60	31.39 (±2.95)	10.65 (±4.36)	48 (80.0)	12 (20.0)	9 (15.0)	1 (1.7)	11 (18.3)	7 (11.7)	0 (0.0)
Manufacturing	33,785	30.40 (±4.11)	11.11 (±4.65)	25,721 (76.1)	8,086 (23.9)	5,056 (15.0)	1,455 (4.3)	5,772 (17.1)	3,674 (10.9)	488 (1.4)
Electricity, gas, steam, and water supply	631	31.51 (±3.40)	13.17 (±4.39)	489 (77.5)	142 (22.5)	92 (14.6)	29 (4.6)	97 (15.4)	91 (14.4)	14 (2.2)
Sewerage, waste management, materials recovery, and remediation activities	144	31.57 (±4.01)	7.22 (±4.28)	108 (75.0)	36 (25.0)	27 (18.8)	1 (0.7)	16 (11.1)	11 (7.6)	0 (0.0)
Construction	6,073	31.84 (±3.93)	7.42 (±4.31)	4,630 (76.2)	1,443 (23.8)	950 (15.6)	238 (3.9)	983 (16.2)	604 (9.9)	84 (1.4)
Wholesale and retail trade	20,539	31.21 (±4.10)	8.92 (±4.67)	15,475 (75.3)	5,074 (24.7)	3,241 (15.8)	863 (4.2)	3,441 (16.8)	2,165 (10.5)	350 (1.7)
Transportation	3,761	31.15 (±3.50)	11.47 (±4.39)	2,934 (78.0)	827 (22.0)	543 (14.4)	150 (4.0)	655 (17.4)	418 (11.1)	73 (1.9)
Accommodation and food service activities	3,864	31.10 (±4.71)	7.47 (±4.47)	2,823 (73.1)	1,042 (27.0)	637 (16.5)	202 (5.2)	610 (15.8)	386 (10.0)	64 (1.7)
Information and communications	6,450	31.31 (±3.69)	10.61 (±4.62)	5,068 (78.6)	1,386 (21.5)	905 (14.0)	245 (3.8)	1,088 (16.9)	762 (11.8)	115 (1.8)
Financial and insurance activities	14,199	31.01 (±3.38)	13.98 (±3.75)	11,299 (79.6)	2,903 (20.4)	1,900 (13.4)	545 (3.8)	2,407 (17.0)	1,713 (12.1)	267 (1.9)
Real estate activities and renting and leasing	4,646	31.46 (±3.86)	9.73 (±4.55)	3,530 (76.0)	1,117 (24.0)	737 (15.9)	191 (4.1)	803 (17.3)	545 (11.7)	78 (1.7)
Professional, scientific, and technical activities	10,070	31.10 (±3.86)	8.90 (±4.53)	7,699 (76.5)	2,373 (23.6)	1,585 (15.7)	409 (4.1)	1,754 (17.4)	1,194 (11.9)	190 (1.9)
Business facilities management and business support services	6,145	30.63 (±4.38)	7.70 (±4.14)	4,532 (73.8)	1,615 (26.3)	1,032 (16.8)	292 (4.8)	1,132 (18.4)	694 (11.3)	78 (1.3)
Public administration and defense	15,648	31.77 (±3.36)	11.09 (±3.30)	12,595 (80.5)	3,057 (19.5)	2,077 (13.3)	542 (3.5)	2,587 (16.5)	1,842 (11.8)	288 (1.8)
Education	36,988	31.55 (±3.67)	9.48 (±4.93)	29,217 (79.0)	7,780 (21.0)	5,215 (14.1)	1,391 (3.8)	6,067 (16.4)	4,144 (11.2)	523 (1.4)
Human health and social work activities	34,918	30.57 (±3.84)	9.31 (±4.50)	26,442 (75.7)	8,495 (24.3)	5,421 (15.5)	1,455 (4.2)	6,497 (18.6)	4,175 (12.0)	642 (1.8)
Arts, sports, and recreation-related services	1,154	31.22 (±4.20)	8.39 (±4.33)	847 (73.4)	308 (26.7)	204 (17.7)	49 (4.2)	219 (19.0)	136 (11.8)	23 (2.0)
Membership organizations, repair, and other personal services	10,042	30.99 (±4.04)	8.37 (±4.28)	7,645 (76.1)	2,398 (23.9)	1,523 (15.2)	455 (4.5)	1,835 (18.3)	1,190 (11.9)	178 (1.8)
Activities of households as employers	458	31.11 (±4.02)	8.61 (±4.13)	354 (77.3)	104 (22.7)	64 (14.0)	22 (4.8)	82 (17.9)	55 (12.0)	4 (0.9)
Activities of extraterritorial organizations and bodies	118	33.76 (±3.75)	12.41 (±4.05)	86 (72.9)	32 (27.1)	25 (21.2)	6 (5.1)	22 (18.6)	12 (10.2)	1 (0.8)
**Total**	430,343	31.10 (±4.21)	11.06 (±4.95)	340,088 (79.0)	90,396 (21.0)	58,609 (13.6)	15,497 (3.6)	63,655 (14.8)	47,194 (11.0)	6,726 (1.6)

SD, standard deviation; IUGR, intrauterine growth restriction

There were a total of 210,576 pregnancies in all working women: 36,988 pregnancies among those working in the education field, 34,918 in the human health and social work field, and 33,785 in the manufacturing field, thus comprising more than half of all pregnancy cases in working women. We also identified 219,767 pregnancies in non-working women. Among these, 177,887 (80.9%) delivered children and 41,942 (19.1%) ended in abortion. The percentage of abortions in each field ranged from 19.5% (public administration and defense) to 27.1% (activities of extraterritorial organizations and bodies, e.g., a foreign embassy officer). The percentage of abortions was higher in all working women (23.0%) than in non-working women (19.1%). Regarding each obstetric outcome, working women had a higher percentage of all adverse obstetric outcomes than non-working women.

We compared women in each industry to dependents of those with employment-based NHI (i.e., the non-working women group) and calculated odds ratios (ORs) for obstetric complications (Table 3).

**Table 3:** Age and income-adjusted odds ratio for abortion and obstetric complications by industry (vs. non-working women).

Industry	Pregnancy	Miscarriage		Threatened abortion		Preterm labor		IUGR	
		N (%)	AOR	N (%)	AOR	N (%)	AOR	N (%)	AOR
**Dependents of those with employment-based NHI** **(non-working women)**	219,767	34,143 (15.5)	1.00 (Ref.)	27,426 (12.5)	1.00 (Ref.)	23,289 (10.6)	1.00 (Ref.)	3,253 (1.5)	1.00 (Ref.)
**All working women**	210,576	39,963 (19.0)	**1.26** **(1.23–1.28)**	36,229 (17.2)	**1.38** **(1.36–1.41)**	23,905 (11.4)	**1.10** **(1.07–1.12)**	3,473 (1.6)	**1.19** **(1.13–1.25)**
Agriculture, forestry, and fishing	402	88 (21.9)	**1.41** **(1.10–1.80)**	60 (14.9)	1.20 (0.91–1.58)	37 (9.2)	0.95 (0.68–1.33)	3 (0.7)	0.57 (0.18–1.78)
Mining and quarrying	60	10 (16.7)	0.89 (0.42–1.89)	11 (18.3)	1.55 (0.81–2.95)	7 (11.7)	1.01 (0.44–2.35)	0 (0.0)	NA
Manufacturing	33,785	6,511 (19.3)	**1.35** **(1.31–1.39)**	5,772 (17.1)	**1.37** **(1.33–1.42)**	3,674 (10.9)	1.02 (0.99–1.06)	488 (1.4)	1.02 (0.92–1.12)
Electricity, gas, steam, and water supply	631	121 (19.2)	**1.26** **(1.03–1.55)**	97 (15.4)	**1.27** **(1.02–1.58)**	91 (14.4)	**1.34** **(1.06–1.68)**	14 (2.2)	1.45 (0.84–2.52)
Sewerage, waste management, materials recovery, and remediation activities	144	28 (19.4)	1.21 (0.79–1.85)	16 (11.1)	0.91 (0.54–1.53)	11 (7.6)	0.78 (0.42–1.45)	0 (0.0)	NA
Construction	6,073	1,188 (19.6)	**1.18** **(1.10–1.26)**	983 (16.2)	**1.31** **(1.23–1.41)**	604 (9.9)	1.02 (0.93–1.11)	84 (1.4)	1.02 (0.82–1.28)
Wholesale and retail trade	20,539	4,104 (20.0)	**1.29** **(1.25–1.34)**	3,441 (16.8)	**1.34** **(1.29–1.39)**	2,165 (10.5)	1.02 (0.97–1.07)	350 (1.7)	**1.24** **(1.11–1.39)**
Transportation	3,761	693 (18.4)	**1.34** **(1.23–1.46)**	655 (17.4)	**1.51** **(1.39–1.65)**	418 (11.1)	1.11 (1.00–1.24)	73 (1.9)	1.30 (1.00–1.68)
Accommodation and food service activities	3,864	839 (21.7)	**1.40** **(1.29–1.52)**	610 (15.8)	**1.28** **(1.17–1.40)**	386 (10.0)	0.98 (0.87–1.09)	64 (1.7)	1.25 (0.97–1.62)
Information and communications	6,450	1,150 (17.8)	**1.12** **(1.04–1.20)**	1,088 (16.9)	**1.36** **(1.27–1.45)**	762 (11.8)	**1.14** **(1.05–1.23)**	115 (1.8)	**1.29** **(1.06–1.56)**
Financial and insurance activities	14,199	2,445 (17.2)	**1.18** **(1.13–1.24)**	2,407 (17.0)	**1.36** **(1.30–1.42)**	1,713 (12.1)	**1.11** **(1.05–1.17)**	267 (1.9)	**1.20** **(1.05–1.37)**
Real estate activities and renting and leasing	4,646	928 (20.0)	**1.28** **(1.19–1.38)**	803 (17.3)	**1.41** **(1.30–1.52)**	545 (11.7)	**1.15** **(1.05–1.26)**	78 (1.7)	1.22 (0.97–1.54)
Professional, scientific, and technical activities	10,070	1,994 (19.8)	**1.29** **(1.22–1.35)**	1,754 (17.4)	**1.39** **(1.31–1.46)**	1,194 (11.9)	**1.16** **(1.09–1.23)**	190 (1.9)	**1.39** **(1.19–1.61)**
Business facilities management and business support services	6,145	1,324 (21.5)	**1.47** **(1.38–1.57)**	1,132 (18.4)	**1.48** **(1.39–1.59)**	694 (11.3)	**1.11** **(1.02–1.20)**	78 (1.3)	0.94 (0.74–1.19)
Public administration and defense	15,648	2,619 (16.7)	**1.08** **(1.02–1.14)**	2,587 (16.5)	**1.34** **(1.27–1.41)**	1,842 (11.8)	**1.16** **(1.09–1.23)**	288 (1.8)	**1.37** **(1.18–1.58)**
Education	36,988	6,606 (17.9)	**1.12** **(1.09–1.16)**	6,067 (16.4)	**1.33** **(1.29–1.38)**	4,144 (11.2)	**1.09** **(1.05–1.13)**	523 (1.4)	1.04 (0.94–1.15)
Human health and social work activities	34,918	6,876 (19.7)	**1.33** **(1.29–1.37)**	6,497 (18.6)	**1.47** **(1.43–1.52)**	4,175 (12.0)	**1.14** **(1.10–1.18)**	642 (1.8)	**1.34** **(1.23–1.46)**
Arts, sports, and recreation-related services	1,154	253 (21.9)	**1.45** **(1.25–1.67)**	219 (19.0)	**1.52** **(1.31–1.77)**	136 (11.8)	1.17 (0.97–1.40)	23 (2.0)	1.55 (1.02–2.35)
Membership organizations, repair, and other personal services	10,042	1,978 (19.7)	**1.27** **(1.20–1.34)**	1,835 (18.3)	**1.44** **(1.37–1.52)**	1,190 (11.9)	**1.18** **(1.10–1.25)**	178 (1.8)	1.33 (1.13–1.55)
Activities of households as employers	458	86 (18.8)	1.18 (0.93–1.50)	82 (17.9)	**1.44** **(1.14–1.83)**	55 (12.0)	1.18 (0.89–1.57)	4 (0.9)	0.65 (0.24–1.75)
Activities of extraterritorial organizations and bodies	118	31 (26.3)	**1.66** **(1.10–2.50)**	22 (18.6)	1.50 (0.94–2.39)	12 (10.2)	0.94 (0.51–1.75)	1 (0.8)	0.57 (0.08–4.06)

IUGR, intrauterine growth restriction; AOR, Age and income adjusted odds ratio; NA, not applicable; Ref., reference; Bold text, statistically significant values

Regarding miscarriage, working women had higher and statistically significant adjusted ORs in 18 out of 21 industries. The age and income-adjusted OR of miscarriage for all working women was 1.26 (95% confidence interval [CI], 1.23–1.28), and 12 industries showed higher values than this. For threatened abortion, all working women groups showed a higher adjusted OR (1.38; 95% CI, 1.36–1.41), and 17 industry groups showed statistically significant adjusted ORs. After excluding industries with fewer than 5,000 pregnancy cases, workers in business facilities management and business support services, manufacturing, human health and social work activities, wholesale and retail trade, and professional, scientific, and technical activities had the five highest adjusted ORs for miscarriage: 1.47 (95% CI, 1.38–1.57), 1.35 (95% CI, 1.31–1.39), 1.33 (95% CI, 1.29–1.37), 1.29 (95% CI, 1.25–1.34), and 1.29 (95% CI, 1.22–1.35), respectively. The adjusted ORs for preterm labor and IUGR are indicated in Table 3.

When comparing each industry to the education field, the results were similar, but the ORs were slightly reduced. Twelve out of 21 industry groups had statistically significantly high adjusted ORs for miscarriage (Table 4).

**Table 4:** Age and income-adjusted odds ratio for abortion and obstetric complications by industry (vs. education).

Industry	Pregnancy	Miscarriage		Threatened abortion		Preterm labor		IUGR	
		N (%)	AOR	N (%)	AOR	N (%)	AOR	N (%)	AOR
**Education**	36,988	6,606 (17.9)	1.00 (Ref.)	6,067 (16.4)	1.00 (Ref.)	4,144 (11.2)	1.00 (Ref.)	523 (1.4)	1.00 (Ref.)
Agriculture, forestry, and fishing	402	39,963 (19.0)	1.23 (0.96–1.57)	60 (14.9)	0.89 (0.68–1.18)	37 (9.2)	0.87 (0.62–1.22)	3 (0.7)	0.57 (0.18–1.78)
Mining and quarrying	60	88 (21.9)	0.81 (0.38–1.72)	11 (18.3)	1.16 (0.61–2.22)	7 (11.7)	0.92 (0.40–2.14)	0 (0.0)	NA
Manufacturing	33,785	10 (16.7)	**1.22** **(1.17–1.27)**	5,772 (17.1)	1.02 (0.98–1.06)	3,674 (10.9)	0.92 (0.88–0.97)	488 (1.4)	0.95 (0.84–1.09)
Electricity, gas, steam, and water supply	631	6,511 (19.3)	1.17 (0.95–1.44)	97 (15.4)	0.95 (0.76–1.19)	91 (14.4)	1.21 (0.96–1.52)	14 (2.2)	1.32 (0.75–2.30)
Sewerage, waste management, materials recovery, and remediation activities	144	121 (19.2)	1.05 (0.69–1.61)	16 (11.1)	0.68 (0.41–1.15)	11 (7.6)	0.73 (0.39–1.34)	0 (0.0)	NA
Construction	6,073	28 (19.4)	1.03 (0.96–1.10)	983 (16.2)	0.99 (0.92–1.07)	604 (9.9)	0.94 (0.86–1.03)	84 (1.4)	1.02 (0.81–1.30)
Wholesale and retail trade	20,539	1,188 (19.6)	**1.14** **(1.09–1.20)**	3,441 (16.8)	1.00 (0.95–1.05)	2,165 (10.5)	0.94 (0.89–0.99)	350 (1.7)	**1.21** **(1.05–1.39)**
Transportation	3,761	4,104 (20.0)	**1.22** **(1.11–1.34)**	655 (17.4)	**1.13** **(1.03–1.24)**	418 (11.1)	1.01 (0.90–1.13)	73 (1.9)	1.21 (0.92–1.59)
Accommodation and food service activities	3,864	693 (18.4)	**1.22** **(1.12–1.33)**	610 (15.8)	0.96 (0.87–1.05)	386 (10.0)	0.90 (0.80–1.01)	64 (1.7)	1.26 (0.96–1.64)
Information and communications	6,450	839 (21.7)	1.01 (0.94–1.08)	1,088 (16.9)	1.01 (0.94–1.09)	762 (11.8)	1.04 (0.95–1.13)	115 (1.8)	1.22 (0.99–1.50)
Financial and insurance activities	14,199	1,150 (17.8)	**1.11** **(1.05–1.17)**	2,407 (17.0)	1.01 (0.96–1.07)	1,713 (12.1)	1.00 (0.93–1.06)	267 (1.9)	1.07 (0.91–1.26)
Real estate activities and renting and leasing	4,646	2,445 (17.2)	**1.15** **(1.06–1.24)**	803 (17.3)	1.06 (0.97–1.15)	545 (11.7)	1.06 (0.96–1.16)	78 (1.7)	1.18 (0.92–1.50)
Professional, scientific, and technical activities	10,070	928 (20.0)	**1.14** **(1.07–1.20)**	1,754 (17.4)	1.04 (0.98–1.10)	1,194 (11.9)	1.06 (0.99–1.14)	190 (1.9)	**1.35** **(1.14–1.61)**
Business facilities management and business support services	6,145	1,994 (19.8)	**1.28** **(1.20–1.37)**	1,132 (18.4)	**1.10** **(1.02–1.18)**	694 (11.3)	1.02 (0.93–1.11)	78 (1.3)	0.94 (0.73–1.21)
Public administration and defense	15,648	1,324 (21.5)	0.98 (0.92–1.04)	2,587 (16.5)	1.01 (0.95–1.07)	1,842 (11.8)	1.06 (0.99–1.13)	288 (1.8)	1.29 1.09–1.53)
Human health and social work activities	34,918	6,876 (19.7)	**1.18** **(1.13–1.23)**	6,497 (18.6)	**1.09** **(1.05–1.13)**	4,175 (12.0)	1.04 (0.99–1.09)	642 (1.8)	**1.30** **(1.15–1.47)**
Arts, sports, and recreation-related services	1,154	253 (21.9)	**1.27** **(1.10–1.48)**	219 (19.0)	1.14 (0.98–1.32)	136 (11.8)	1.07 (0.89–1.29)	23 (2.0)	1.53 (1.00–2.34)
Membership organizations, repair, and other personal services	10,042	1,978 (19.7)	**1.11** **(1.05–1.18)**	1,835 (18.3)	**1.08** **(1.01–1.14)**	1,190 (11.9)	1.08 (1.01–1.16)	178 (1.8)	**1.31** **(1.10–1.56)**
Activities of households as employers	458	86 (18.8)	1.04 (0.82–1.32)	82 (17.9)	1.08 (0.85–1.37)	55 (12.0)	1.08 (0.81–1.44)	4 (0.9)	0.64 (0.24–1.72)
Activities of extraterritorial organizations and bodies	118	31 (26.3)	**1.54** **(1.02–2.32)**	22 (18.6)	1.16 (0.73–1.85)	12 (10.2)	0.87 (0.47–1.61)	1 (0.8)	0.52 (0.07–3.75)

IUGR, intrauterine growth restriction; AOR, Age and income adjusted odds ratio; NA, not applicable; Ref., reference; Bold text, statistically significant values

The order of the highest adjusted ORs for miscarriage did not change much, and the top five industries with more than 5,000 pregnancy cases also had the same order: 1.28 (95% CI, 1.20–1.37), 1.22 (95% CI, 1.17–1.27), 1.18 (95% CI, 1.13–1.23), 1.14 (95% CI, 1.09–1.20), and 1.14 (95% CI, 1.07–1.20) for business facilities management and business support services, manufacturing, human health and social work activities, wholesale and retail trade, and professional, scientific, and technical activities, respectively. The adjusted ORs for other obstetric complications are shown in Table 4. Only 3 out of 21 industry groups had high adjusted ORs; ORs were 1.47 (95% CI, 1.08–2.00) for fetal screening abnormalities in transportation workers, and 1.44 (95% CI, 1.25–1.66) and 1.41 (95% CI, 1.15–1.72) in human health and social work activities workers, and public administration and defense workers, respectively. Adjusted ORs for other obstetric complications are shown in Table 4. No industries had higher adjusted ORs for preterm labor. Wholesale and retail trade, professional, scientific, and technical activities, human health and social work activities, and membership organizations, repair, and other personal services had higher adjusted ORs for IUGR: 1.21 (95% CI, 1.05–1.39), 1.35 (95% CI, 1.14–1.61), 1.30 (95% CI, 1.15–1.47), and 1.31 (95% CI, 1.10–1.56), respectively.

## Discussion

Our study results showed that work activities *per se* can negatively affect maternal health. In a comparison of all working women and non-working women, miscarriage, threatened abortion, preterm labor, and IUGR had higher adjusted ORs, which were statistically significant in working women. This study was performed to assess the risk of work activity as a whole, not the specific work. Many studies have focused on specific agents, but few studies have mainly focused on the work activity itself. Lemasters et al. [35], after analyzing occupational exposures and spontaneous abortion, suggested that maternal employment status could play a role as a confounder. Their study showed a higher relative risk (RR) for spontaneous abortion during employment. Interestingly, their RR of 1.23 (95% CI, 1.02–1.49) was very close to our OR for miscarriage (1.26; 95% CI, 1.23–1.28). Our results support the general tendencies of risk for maternal health in working women.

There are several major industries that place women at high risk for adverse obstetric outcomes; business facilities management and business support services, manufacturing, human health and social work activities, wholesale and retail trade, and professional, scientific, and technical activities were shown to be the high-risk industry groups (Table 3). In addition, there are many suspicious reprotoxic agents in various work environments.

Business facilities management and business support services also comprise various jobs such as cleaning and maintaining buildings, landscape care, and travel agents. Several ways of exposure to reprotoxic agents exist, such as physical labor, irregular work times, and cleaning and gardening with various chemicals and pesticides. It was difficult to attribute risk to specific agents because of the limited data and the study design. Manufacturing has 32 groups and 76 classes of industries. We conducted a sub-analysis within each manufacturing industry group, and several industries were shown to have higher risks (data not shown). According to the results of the sub-analyses, further studies focusing on manufacturing work involving electronic devices, semiconductors, and chemicals are required. Lead in battery manufacturing [36], as well as ethylene, styrene, and propylene in plastic manufacturing are reported to be reprotoxic chemicals [27]. Manufacturing electronic devices has been studied in many groups with regard to exposure to chemicals. Kim et al. [33] described increased RRs for spontaneous abortion (RR = 1.57) and menstrual aberration (RR = 1.54) among workers in major Korean semiconductor companies. Their results were driven by NHI claim data for 5 consecutive years. However, their study design was slightly different from ours; they aggregated data for 5 years and did not consider individuals’ socio-economic status. More extensive studies involving semiconductor workers are needed because of the risks involved with their jobs, work places, work periods, and work durations.

Among the five major industries, workers involved in human health and social work activities were relatively well represented. Doctors, nurses, radiation workers, and other health care providers comprise this group. However, in comparison with non-working women, they had higher ORs for miscarriage, threatened abortion, preterm labor, and IUGR. Similar results were shown in comparison to education workers, but slightly reduced adjusted ORs were observed. Regarding the adverse reproductive outcomes in health care workers, specifically nurses, an intensive evaluation is needed.

The professional, scientific, and technical activities group comprises laboratory workers who are exposed to chemicals, solvents, bacteria, and radioisotopes while performing laboratory work [37]. Several studies regarding various reproductive outcomes for laboratory workers have been performed. Halliday-Bell et al. [38] conducted a cohort study involving laboratory workers in Finland; the RR for low birth weight was 1.27 (95% CI, 1.08–1.45) compared to teachers. Regarding IUGR, we also compared education workers as a reference, and our adjusted OR was 1.35 (95% CI, 1.14–1.61), which is similar to our data in terms of the reference group and risk values.

Our study has several limitations. The classification of study populations was based only on codes of NHI data. An incorrect diagnosis or erroneous codes could have been entered, even though NHI has a surveillance organization. Specifically, pregnancy losses or delivery outcomes seem reliable because they are difficult to misdiagnose. However, differentiating among threatened abortions, fetal growth retardation, and preterm labor based on codes may be relatively unreliable. This limitation was inevitable because we used secondary data, which also has characteristics of big data. We consider this as an important limitation, but the probability that a certain population or industry group had a more differential distribution of coding errors or misclassification seems low.

Another limitation was the exposure assessment, which is crucial in research on occupational environments. An evaluation of specific reprotoxic agents, their exposure time, route, type, concentration, or intensity would be informative. However, we were unable to obtain information in each industry’s work environment. Therefore, the results may not well represent the characteristics of the work environment in certain industries’ or of certain working women. Additionally, the results of an adverse pregnancy outcome may not be attributable to a specific work environment.

Since NHI claim data did not include each individual’s duration of work and our study design was cross-sectional (not a cohort study), we were unable to consider individual durations of work and the time interval between work and pregnancy for our analysis. Therefore, the industry itself may not be the main cause of adverse reproductive outcomes; various other conditions could be attributed. In NHI claim data, only industrial information was provided, not the insured’s job in that field of work. Moreover, industry is the largest classification term and includes various types of jobs. Therefore, our results may not represent specificities of jobs or work environments. However, considering that industries have relatively low adjusted ORs for reproductive outcomes, they can sufficiently reflect job characteristics. For example, financial and insurance activities, education, information and communications, and public administration and defense industries had the lowest adjusted ORs for miscarriage. Bankers, teachers, secretaries, and public officers are represented in these industries, and in general, they are not exposed to reprotoxic chemicals or work in odd shifts. However, our results indicated that even “unexposed” workers in “safe” industries had higher ORs than non-working women, thus supporting the idea that work activities *per se* can negatively affect one’s reproductive health. These results may be used to quantitatively distinguish the adverse effects of “work in general” and reprotoxic agents in the work environment.

Regarding obstetric outcomes, one’s obstetric history is one of the most important factors to consider; however, NHI claim data only includes records of each visit, but not the medical history of each individual. In addition, information regarding alcohol use, smoking status, body mass index, fertility medication use, underlying diseases such as diabetes and hypertension were unavailable. However, we believe that those factors randomly affect each group, and the overall effect is not believed to be significantly different; therefore, the overall results would not change substantially.

Our study revealed the descriptive epidemiology of maternal health in working women and indicated several industries that place women at risk for adverse reproductive outcomes. Only a few studies have reported the descriptive epidemiology of maternal health based on analysis of a large amount of data. We could obtain statistical power by collecting a large amount of data provided by the NHI; our study population included more than 430,000 women who underwent pregnancy, and the study outcomes were based on approximately 80% of pregnancies during 2013. However, because of the large study population, slight differences or insignificant results could have been considered statistically significant. We recommend interpreting the study results while considering the study populations and CIs.

In conclusion, we confirmed that working women are at risk for adverse pregnancy outcomes such as miscarriage, threatened abortion, preterm labor, and IUGR. Our study provides incidence and distribution of adverse obstetric outcomes, suggesting that several industries need maternal health protection and development of other protective measures in future. We suggest further investigations regarding reprotoxic hazards in specific populations to resolve maternal health inequalities among working women.
