# Correction: Prevalence of abortion and adverse pregnancy outcomes among working women in Korea: A cross-sectional study

**DOI:** 10.1371/journal.pone.0188673

**Published:** 2017-11-20

**Authors:** Chulyong Park, Mo-Yeol Kang, Dohyung Kim, Jaechan Park, Huisu Eom, Eun-A Kim

Affiliation 2 for author Chulyong Park is incomplete. Affiliation 2 should be: Department of Occupational and Environmental Medicine, Samsung Changwon Hospital, Sungkyunkwan University School of Medicine, Changwon, Korea.
